# Effect of Metal Primer Application on the Shear Bond Strength of Orthodontic Brackets with Different Base Designs: An In Vitro Study **

**DOI:** 10.3390/dj14090581

**Published:** 2026-09-10

**Authors:** Abdeen Shaker, Selim Arici

**Affiliations:** Faculty of Dentistry, Atlas University, Istanbul 34408, Türkiye

**Keywords:** orthodontic bonding, metal primer, shear bond strength, bracket base design, adhesive remnant index, enamel integrity

## Abstract

**Background/Objectives:** Direct bonding of orthodontic brackets is standard in fixed-appliance therapy, but clinical success depends on achieving reliable bond strength without compromising enamel integrity during debonding. This in vitro study evaluated whether applying a metal primer to stainless-steel bracket bases affects shear bond strength (SBS) to enamel, and whether primer coverage and bracket-base design influence adhesive-remnant distribution and enamel integrity. **Methods:** A total of 132 extracted human maxillary premolars were randomly allocated to six groups (n = 22 each) defined by bracket base design (mesh or integral) and primer coverage (none, full, or partial central coverage). Brackets were bonded with Transbond XT, and Reliance Metal Primer was applied according to group allocation. After 2000 thermal cycles (5–55 °C), specimens were debonded in shear at 1 mm/min. SBS was calculated, adhesive remnants were quantified with ImageJ and scored using the adhesive remnant index (ARI), and enamel damage was assessed using an enamel damage index (EDI). **Results:** Mean SBS values were 15.68 MPa (MC), 18.38 MPa (MF), 20.89 MPa (MP), 13.04 MPa (IC), 17.11 MPa (IF), and 12.66 MPa (IP). Two-way ANOVA showed significant main effects of bracket base design (F(1, 126) = 16.06, *p* < 0.001, partial η^2^ = 0.113) and primer coverage (F(2, 126) = 3.98, *p* = 0.021, partial η^2^ = 0.059), together with a significant bracket-base-design × primer-coverage interaction (F(2, 126) = 4.46, *p* = 0.014, partial η^2^ = 0.066). ARI distributions differed significantly across the six experimental groups (χ^2^(15) = 75.24, *p* < 0.001, Cramér’s V = 0.436), whereas EDI scores did not differ significantly among groups (*p* = 0.756). **Conclusions:** The effect of the metal primer on SBS depended on bracket base design and primer coverage. Partial coverage produced the highest SBS among mesh-base brackets and was significantly higher than the mesh control. Among integral-base brackets, full coverage yielded the highest numerical SBS, but within-design pairwise differences were not significant. ARI failure patterns also varied significantly across the six experimental groups, whereas no significant difference in stereomicroscopically detectable enamel damage was observed.

## 1. Introduction

Direct bonding of orthodontic brackets is a fundamental procedure in fixed orthodontic therapy [1]. Compared with conventional banding, direct bonding improves esthetics and patient comfort, facilitates oral hygiene, and may reduce the risk of enamel decalcification and caries when adequate hygiene is maintained [2]. Despite these advantages, clinical success depends on achieving a bracket–enamel bond that is strong enough to resist orthodontic and masticatory forces, because bond failure necessitates rebonding, prolongs treatment, increases costs, and inconveniences both patients and clinicians [3].

Bonding performance is influenced by several factors, including enamel characteristics, adhesive composition, surface treatment, bracket base design, and bracket material [3,4,5,6]. Bonding materials have also been developed to address specific clinical challenges, including moisture sensitivity [7] and antibacterial activity [8]. However, increasing bond strength should not compromise enamel integrity during debonding, because enamel cracks, increased surface roughness, and residual adhesive can adversely affect esthetics and long-term dental health [9,10,11]. Metal primers improve bonding to metallic surfaces by enhancing resin wetting and promoting chemical interaction with the surface oxide layer. Functional monomers such as VBATDT, MMA, 4-META, and 10-MDP (MDP) have been associated with increased shear bond strength on metal substrates [12,13]. This is clinically relevant because bonding to amalgam and other restorative or non-enamel surfaces remains challenging, and surface modification combined with functional-monomer primers can improve interfacial compatibility [14].

Although metal primers are well-documented for bonding to metallic restorations, evidence regarding how the primer-coverage pattern interacts with bracket-base morphology on sound enamel remains limited. Recent in vitro studies continue to show that bracket-base geometry and retentive design can alter SBS and ARI patterns [15,16,17]. More broadly, a 2025 systematic review found lower clinical bracket-failure rates when a conventional orthodontic primer step was used than when it was omitted [18]; however, those findings concern enamel-bonding primers and should not be extrapolated directly to metal primers applied to bracket bases. Therefore, this in vitro study aimed to evaluate whether applying a metal primer to stainless-steel bracket bases affects SBS, adhesive-remnant distribution, and enamel integrity, with particular emphasis on primer coverage (full versus partial) and bracket-base design (mesh versus integral). The null hypothesis was that metal primer application, primer-coverage pattern, and bracket-base design would not significantly affect SBS, ARI, or EDI.

## 2. Materials and Methods

### 2.1. Ethical Approval and Study Design

Ethical approval for the use of extracted human teeth was granted by the Clinical Research Ethics Committee of the Faculty of Dentistry, Ondokuz Mayıs University (approval no. 2019-474; decision date 13 June 2019). The 132 extracted maxillary premolars were randomly allocated to six experimental groups (n = 22 per group) using a simple lottery (drawing-of-lots) method. After A.S. applied the metal primer to the brackets assigned to the respective groups, S.A. coded the groups together with the corresponding teeth and brackets and sealed the allocation codes in an opaque envelope. A.S., who subsequently performed the bonding procedures and all experimental measurements, remained blinded to group allocation throughout the experimental phase. After all measurements and data collection were completed, the sealed envelope was opened, the allocation codes were revealed, and the data were matched to the corresponding specimens before statistical analysis.

### 2.2. Sample-Size Estimation

The sample size was based on the study by Cal Neto et al. [19]. An a priori sample-size calculation was performed using the Statulator power-analysis program, assuming a standard deviation of 6 MPa and a 95% confidence level. The analysis indicated that a minimum of 19 teeth per group was required. To compensate for potential specimen loss during the experimental procedures, the sample size was increased to 22 teeth per group. Consequently, 132 extracted maxillary premolars were included in the study.

### 2.3. Tooth Selection and Storage

A total of 132 human maxillary premolars extracted for orthodontic reasons were included, and no tooth had been stored for more than six months before testing. Maxillary premolars were selected because they are frequently available following orthodontic extraction and because restricting the experiment to a single tooth category reduces substrate-related variability; recent SBS studies have likewise used human premolars for controlled bracket-bond testing [15,17]. Teeth with caries, restorations, cracks, surface defects, previous bonding, endodontic treatment, extraction-related crown damage, or irregular buccal surfaces were excluded. After extraction, the teeth were cleaned and stored at room temperature in 0.1% thymol solution until use.

### 2.4. Brackets, Materials, and Experimental Groups

Two stainless-steel bracket base designs were evaluated: mesh-base and integral (foil/solid) base brackets. The mesh-base brackets were 3M Victory Series MBT metal brackets (3M Unitek, Monrovia, CA, USA; slot size 0.022 inch), with a base area of 9.09 mm^2^ and a micro-etched 80-gauge mesh base. The integral-base brackets were Adenta CROWN™ MBT metal brackets (Adenta GmbH, Gilching, Germany; slot size 0.022 inch), with a base area of 12.64 mm^2^ and a micro-etched mechanical-undercut integral base. Enamel was etched using 34% phosphoric acid gel (Scotchbond™ Universal Etchant, 3M ESPE, Monrovia, CA, USA). Brackets were bonded using Transbond™ XT Light Cure Adhesive Primer and Transbond™ XT adhesive (3M Unitek, Monrovia, CA, USA). Reliance Metal Primer (Reliance Orthodontic Products Inc., Itasca, IL, USA) was used as the metal primer. Polymerization was performed using an Elipar S10 LED curing light (3M ESPE, St. Paul, MN, USA; 1200 mW/cm^2^, 430–480 nm). Thermal aging was performed using a thermocycling device (SALUBRIS-technica, Istanbul, Türkiye).

Transbond™ XT was selected as the adhesive system because it is a widely used light-cured orthodontic composite that has served as a reference material in laboratory SBS studies, including recent investigations of metal bracket-base design [15,16,17]. Reliance Metal Primer was selected because metal-conditioning primers containing functional monomers have been used to improve resin bonding to dental alloys, and previous orthodontic studies have reported enhanced bond strength after metal-surface priming [12,19,20,21]. A single primer–adhesive combination was intentionally used to isolate the effects of bracket-base design and primer-coverage pattern; consequently, the findings may be material-specific and should not be generalized automatically to other primer or adhesive systems.

Teeth were randomly assigned to six groups of 22 specimens each, defined by base design and primer coverage, as summarized in Table 1.

### 2.5. Bonding, Thermocycling, and Debonding

Before bracket bonding, metal primer was applied to the bracket bases according to the assigned experimental group. The control groups received no metal primer, whereas the experimental groups received either full-base or central partial primer coverage. The full-versus-partial comparison was a predefined experimental contrast designed to determine whether limiting the primer-treated portion of the bracket base could modify SBS and failure behavior; partial coverage was not a manufacturer-recommended application technique.

Reliance Metal Primer was applied using a bonding microbrush with a tip diameter of approximately 1 mm. The microbrush was loaded with primer, and excess material was removed by gently touching the brush tip to sterile gauze before application. For the full-coverage groups, primer was distributed over the entire bracket base. For the partial-coverage groups, the microbrush was held perpendicular to the bracket base and gently brought into contact with the visually estimated center of the base. The treated central region was not geometrically measured; however, based on the microbrush-tip diameter and perpendicular contact technique, it was estimated to correspond approximately to a circular area 1 mm in diameter. After application, the primer was left undisturbed on the bracket base for approximately 40 s and then gently air-dried with oil- and water-free air before adhesive application. The amount of primer was standardized indirectly, through consistent microbrush size and excess-removal technique, rather than by volumetric measurement.

After primer application and subsequent coding, A.S. performed the bonding procedures while blinded to group allocation. After enamel etching with 34% phosphoric acid for 30 s, the teeth were rinsed for 30 s, air-dried, and coated with Transbond™ XT Light Cure Adhesive Primer. Transbond™ XT adhesive was then applied to the bracket base, and each bracket was seated on the buccal enamel by the same operator using a consistent clinical placement procedure. Excess adhesive extruded around the bracket margins was removed with a probe before polymerization. No dedicated positioning jig was used, and adhesive-layer thickness was not measured directly. Light curing was performed using an Elipar S10 LED curing light at an irradiance of 1200 mW/cm^2^. According to the manufacturer’s technical specifications, the unit operates over a wavelength range of 430–480 nm, with peak emission at 455 nm. Each bracket was light-cured for 20 s: 10 s from the occlusal aspect and 10 s from the gingival aspect.

Following bonding, the specimens were stored in distilled water at room temperature for 12 h and then subjected to 2000 thermal cycles between 5 °C and 55 °C using a thermocycling device, with a dwell time of 10 s in each bath and immediate transfer between baths. The 5–55 °C range was selected to simulate commonly used thermal extremes in laboratory models of oral temperature change, whereas 2000 cycles provided an artificial-aging challenge before mechanical testing. Thermocycling remains a contemporary approach for assessing aging-related changes in orthodontic bracket bonding and enamel response [22]. For mechanical testing, the crowns were embedded in dental stone (Durguix Pro, Protechno, Spain) within plastic rings, with the occlusal plane positioned horizontally. Debonding was performed using a universal testing machine (Lloyd Instruments Plc, Fareham, Hampshire, UK). The loading blade was positioned beneath the bracket wings and aligned parallel to the bracket base; shear force was applied at a crosshead speed of 1 mm/min until bond failure occurred. Peak debonding force was recorded in Newtons and converted to MPa using the corresponding bracket-base area.

### 2.6. Outcome Assessment

Shear bond strength (SBS, MPa) was calculated by dividing the debonding force (N) by the bracket-base area (mm^2^). Buccal enamel surfaces were photographed under a stereomicroscope (×10) at three time points: before bonding, after debonding, and after removal of residual adhesive. Adhesive remnants were analyzed with ImageJ 1.52p (NIH, Bethesda, MD, USA) to estimate the percentage of adhesive remaining and to assign adhesive remnant index (ARI) scores as follows: 0 = no adhesive left on the tooth; 1 = less than half of the adhesive left on the tooth; 2 = more than half of the adhesive left on the tooth; and 3 = all adhesive left on the tooth. Enamel integrity was screened using a stereomicroscope-based enamel damage index (EDI), defined as follows: 0 = no detectable material loss on the buccal enamel surface; 1 = detectable material loss after debonding and adhesive clean-up. All ARI/EDI scoring and ImageJ measurements were performed by the same examiner (A.S.). Formal intra-examiner repeatability and inter-examiner reliability testing were not performed.

### 2.7. Statistical Analysis

Data were analyzed using IBM SPSS Statistics for Windows, Version 21.0 (IBM Corp., Armonk, NY, USA). The normality of continuous variables was assessed using the Shapiro–Wilk test, and homogeneity of variances was evaluated using Levene’s test. Shear bond strength was analyzed using two-way analysis of variance (ANOVA) to evaluate the main effects of bracket base design (mesh vs. integral), primer coverage (control, full, and partial), and their interaction. When significant differences were detected, Tukey’s honestly significant difference (HSD) test was used for post hoc pairwise comparisons. ARI distributions were compared across all six experimental groups using the Pearson chi-square test. Because several expected cell counts were below 5, the Pearson result was additionally evaluated using a Monte Carlo conditional test based on 100,000 randomly generated contingency tables with fixed marginal totals. The strength of the association was quantified using Cramér’s V, and a 95% confidence interval was estimated by nonparametric bootstrap resampling (20,000 iterations). EDI distributions across the six groups were analyzed using the chi-square test. Quantitative variables are presented as mean ± standard deviation, and categorical variables as frequencies and percentages. Statistical significance was set at *p* < 0.05.

## 3. Results

### 3.1. Shear Bond Strength

**Figure 1:**
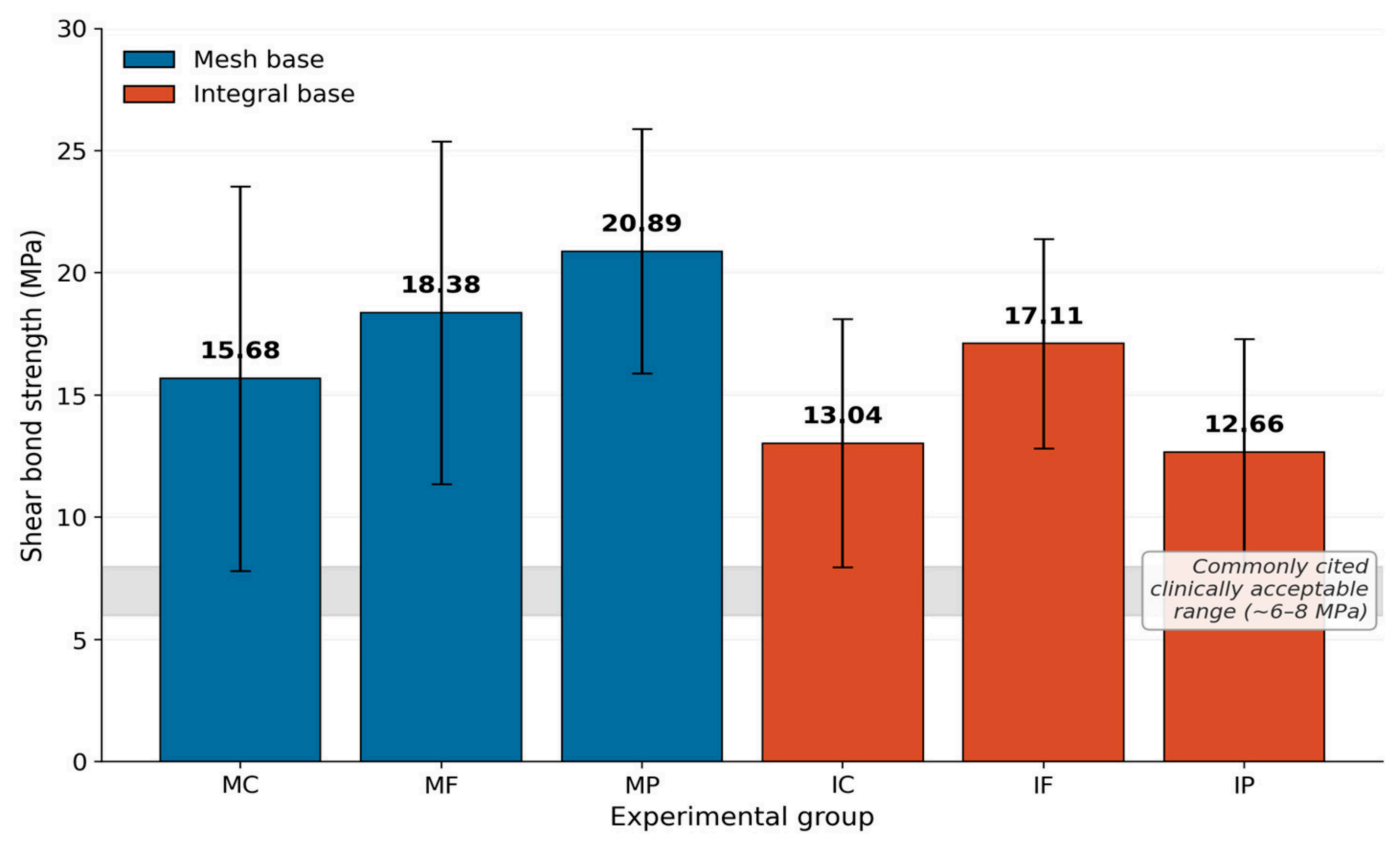
Comparison of mean shear bond strength (SBS) among the six experimental groups. Bars represent mean values, and error bars represent standard deviations. The shaded band denotes the commonly cited clinically acceptable range (~6–8 MPa).

Descriptive SBS statistics are summarized in Table 2. The highest mean SBS was recorded in the mesh-base partial-coverage group (MP, 20.89 ± 5.00 MPa), followed by the mesh-base full-coverage group (MF, 18.38 ± 7.01 MPa) and the integral-base full-coverage group (IF, 17.11 ± 4.29 MPa). The control groups showed intermediate values (MC, 15.68 ± 7.86 MPa; IC, 13.04 ± 5.07 MPa), whereas the lowest mean SBS was observed in the integral-base partial-coverage group (IP, 12.66 ± 4.65 MPa). Two-way ANOVA demonstrated a significant main effect of bracket base design (F(1, 126) = 16.06, *p* < 0.001, partial η^2^ = 0.113), indicating higher SBS values for mesh-base than for integral-base brackets when averaged across primer-coverage conditions. A significant main effect of primer coverage was also observed (F(2, 126) = 3.98, *p* = 0.021, partial η^2^ = 0.059). Furthermore, the bracket-base-design × primer-coverage interaction was significant (F(2, 126) = 4.46, *p* = 0.014, partial η^2^ = 0.066), indicating that the effect of metal primer on SBS depended on bracket-base design, as shown in Figure 1.

Post hoc Tukey HSD comparisons showed that, among mesh-base brackets, partial primer coverage produced significantly higher SBS than the control condition (MP vs. MC: mean difference = 5.22 MPa, 95% CI: 0.16–10.27, *p* = 0.039), whereas full coverage did not differ significantly from either the control or partial-coverage condition. Among integral-base brackets, no statistically significant differences were detected among the control, full-coverage, and partial-coverage groups. Cross-base comparisons showed significantly higher SBS for MF than for IC and IP and for MP than for IC and IP (all *p* < 0.05). Detailed pairwise comparisons are presented in Table 3.

### 3.2. Adhesive Remnant Index

**Figure 2:**
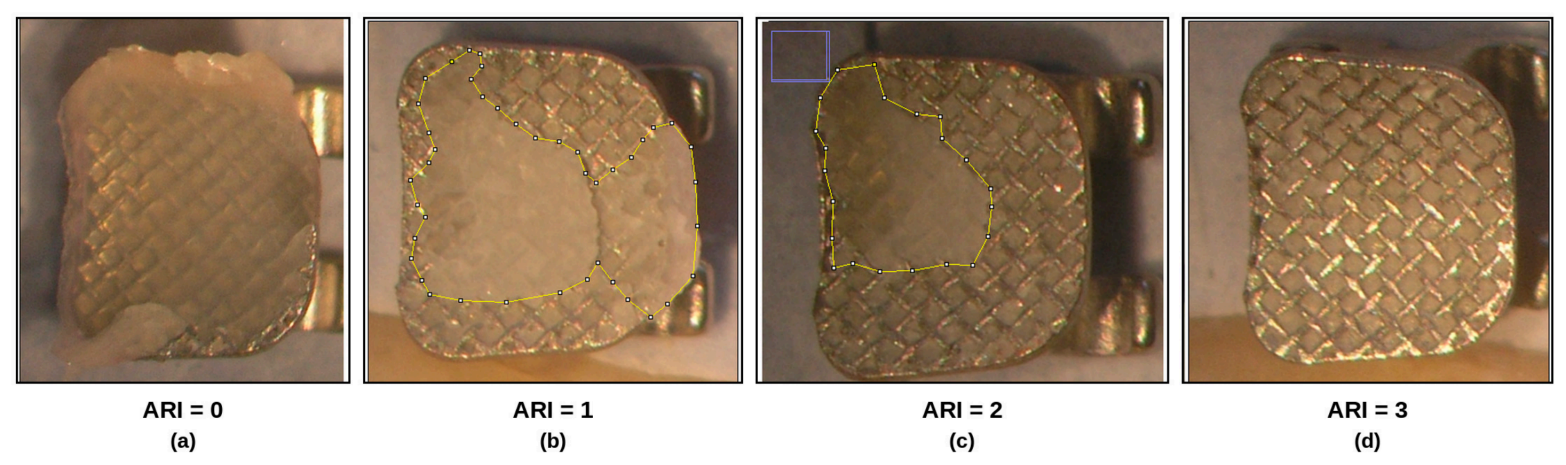
Representative stereomicroscopic images (×10) used for adhesive remnant index (ARI) scoring: (**a**–**d**) increasing amounts of adhesive remaining on the enamel surface after debonding.

**Figure 3:**
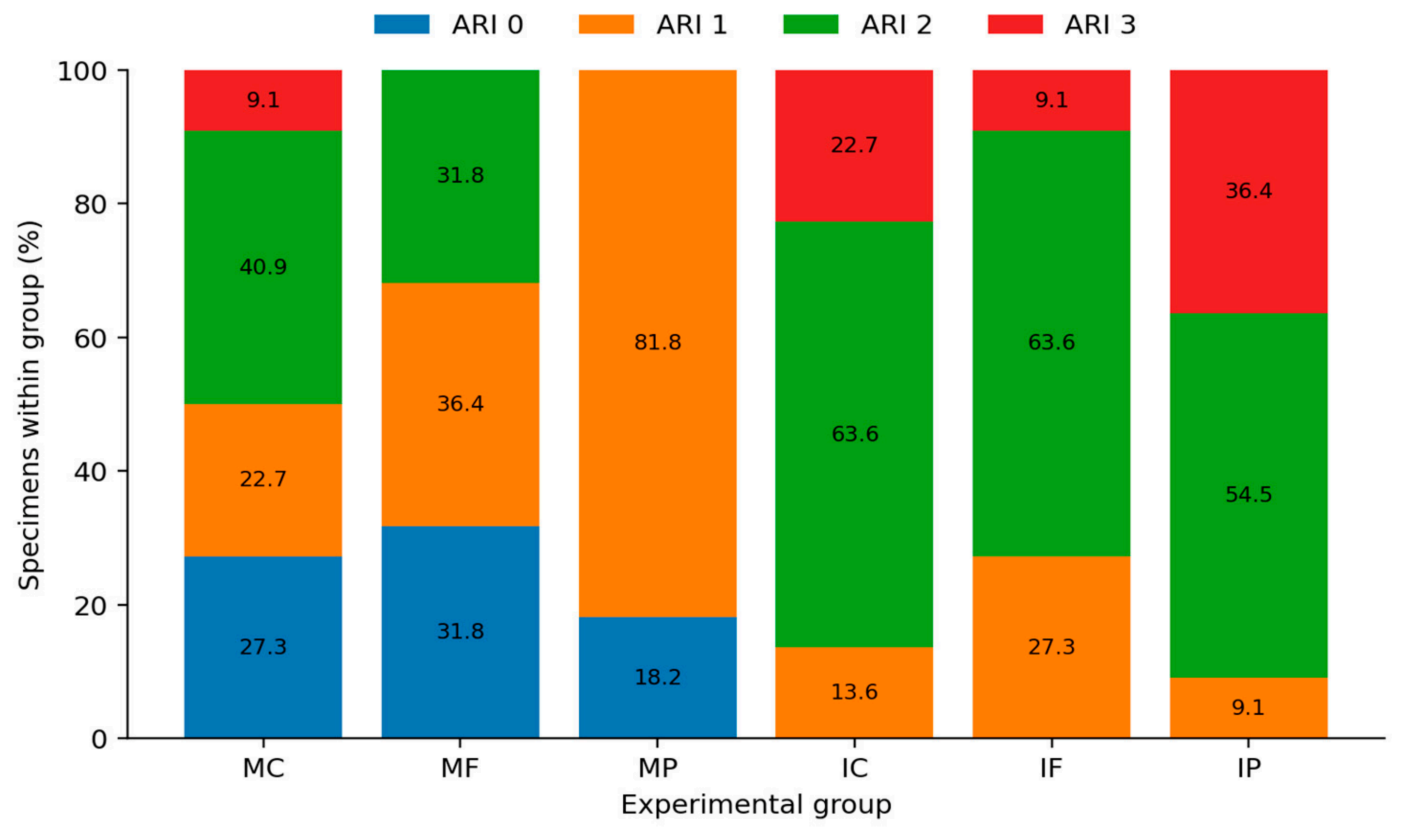
Distribution of ARI scores among the six experimental groups, expressed as the percentage of specimens within each group.

ARI distributions differed significantly across the six experimental groups (Pearson χ^2^(15) = 75.24, *p* < 0.001; Monte Carlo *p* < 0.001; Cramér’s V = 0.436, 95% bootstrap CI: 0.401–0.539; Table 4), indicating a moderate-to-large association between experimental group and failure pattern. In the mesh partial-coverage (MP) group, ARI score 1 predominated (18/22; 81.8%), whereas the integral-base groups showed predominantly higher ARI scores. Specifically, ARI score 2 accounted for 63.6% of both IC and IF specimens and 54.5% of IP specimens, whereas ARI score 3 was most frequent in IP (8/22; 36.4%). These findings indicate that adhesive-remnant distribution varied not only with bracket-base design but also across primer-coverage conditions. The Monte Carlo conditional analysis confirmed that the overall association remained statistically significant despite the sparse expected counts. Representative stereomicroscopic images used for ARI scoring are shown in Figure 2, and the six-group score distribution is presented in Figure 3.

### 3.3. Enamel Damage Index

**Figure 4:**
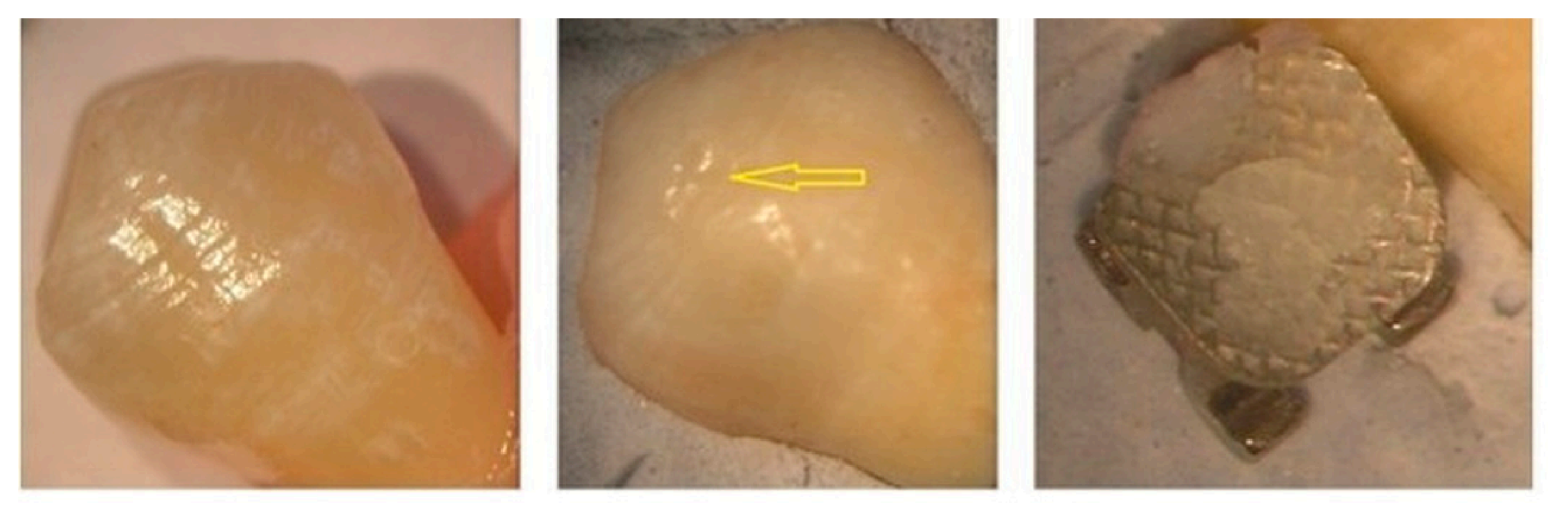
Representative stereomicroscopic images used for enamel damage index (EDI) evaluation, showing the buccal enamel surface before and after debonding and adhesive clean-up.

**Figure 5:**
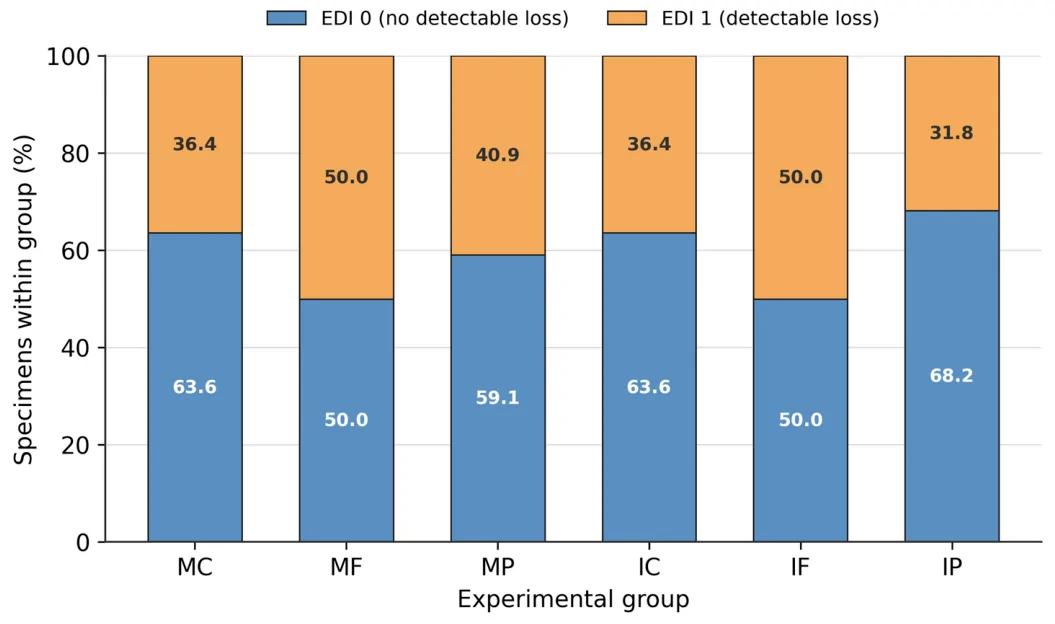
Distribution of EDI scores among the six experimental groups, expressed as the percentage of specimens within each group.

No statistically significant difference in EDI scores was detected among the groups (χ^2^ test, *p* = 0.756; Table 5). In all groups, most specimens showed no stereomicroscopically detectable material loss after debonding and standardized adhesive removal (Figure 4 and Figure 5).

## 4. Discussion

This study evaluated the influence of metal primer application on shear bond strength (SBS), adhesive-remnant distribution, and enamel-surface integrity in orthodontic brackets with two different base designs. Factorial analysis demonstrated significant main effects of bracket-base design and primer coverage, together with a significant interaction between these factors. Accordingly, the effect of metal primer on SBS cannot be interpreted independently of bracket-base morphology, and the null hypothesis was partially rejected.

Bracket-base design showed a significant main effect on SBS (F(1, 126) = 16.06, *p* < 0.001, partial η^2^ = 0.113). When averaged across primer-coverage conditions, mesh-base brackets showed a higher mean SBS than integral-base brackets (18.31 vs. 14.27 MPa). This finding supports the view that bracket-base morphology contributes to bonding performance [5,6,15]. Contemporary evidence is consistent with the broader principle that retentive geometry matters: Esmail et al. reported significant SBS differences among bracket-base shapes together with variation in ARI scores [16], whereas Duran et al. found that changes in the retentive base design of 3D-printed brackets altered both SBS and adhesive-retention patterns [17]. However, the direction and magnitude of these effects vary among specific bracket systems, materials, and base geometries. In the present study, the nonsignificant Tukey comparison between the two control groups (MC vs. IC, *p* = 0.661) further indicates that the overall main effect of base design should not be interpreted as a uniform difference under every primer condition.

Primer coverage also showed a significant main effect (F(2, 126) = 3.98, *p* = 0.021, partial η^2^ = 0.059), but this effect was modified by bracket-base design, as demonstrated by the significant bracket-base-design × primer-coverage interaction (F(2, 126) = 4.46, *p* = 0.014, partial η^2^ = 0.066). This interaction is central to interpreting the present findings: metal primer did not exert a uniform effect across the two bracket designs. Instead, the response to primer application varied according to both the extent of primer coverage and the retentive morphology of the bracket base. Metal primers may enhance the resin–metal interface by improving wetting and promoting chemical interactions between functional monomers and the oxide layer on metallic surfaces [12,21]. Al Taweel et al. likewise reported increased orthodontic bracket bond strength after bracket-surface priming [20]. Nevertheless, the present experiment did not directly measure surface chemistry, primer penetration, or interfacial morphology; therefore, these mechanisms remain explanatory hypotheses rather than demonstrated pathways.

Previous investigations have largely evaluated bracket-surface priming [20] and bracket-base-design effects [15] as separate questions. The novelty of the present study lies in evaluating primer-coverage pattern and bracket-base design within the same factorial framework. Rather than assessing metal-primer application as an isolated intervention, the present findings show that its effect on SBS depends on the interaction between primer coverage and bracket-base morphology. In addition, the combined assessment of SBS, ARI, and enamel damage provides complementary information regarding bond strength, failure pattern, and enamel safety.

Within the mesh-base groups, mean SBS increased from 15.68 MPa in the control group (MC) to 18.38 MPa with full coverage (MF) and 20.89 MPa with partial central coverage (MP). Tukey HSD testing confirmed that MP had significantly higher SBS than MC (mean difference = 5.22 MPa, 95% CI: 0.16–10.27, *p* = 0.039), whereas MF did not differ significantly from either MC or MP. Thus, partial primer coverage was associated with the highest SBS in the mesh-base system and was the only primer condition that significantly exceeded the mesh control. One possible explanation is that localized primer application was sufficient to modify part of the metal–resin interface while leaving the surrounding mesh available for direct mechanical interlocking with the adhesive. Conversely, full coverage may have altered the relative contribution of these two retention pathways. These possibilities were not directly tested and should therefore be regarded as hypotheses. This also helps explain the mechanistic relevance of comparing full versus central coverage: the extent of bracket-base surface exposed to primer may interact with base architecture rather than produce a simple dose–response effect.

A different numerical pattern was observed for integral-base brackets. Mean SBS was 13.04 MPa in the control group (IC), increased to 17.11 MPa with full primer coverage (IF), and was 12.66 MPa with partial coverage (IP). Nevertheless, the within-integral pairwise comparisons were not statistically significant (IC vs. IF, *p* = 0.192; IC vs. IP, *p* = 1.000; IF vs. IP, *p* = 0.120). Therefore, although full coverage produced the highest numerical SBS within the integral-base design, it should not be described as statistically superior to the other integral-base conditions. Unlike an open mesh, the integral base provides a continuous micro-etched mechanical-undercut surface; consequently, a localized central primer layer may interact differently with the available retentive features. The low mean observed with partial coverage may suggest that limited application is less favorable for this particular integral-base system, but this interpretation remains tentative and should be confirmed by direct microscopic or chemical assessment and by studies using additional bracket designs.

The revised six-group ARI analysis demonstrated a significant association between experimental group and adhesive-remnant pattern (χ^2^(15) = 75.24, *p* < 0.001; Cramér’s V = 0.436, 95% bootstrap CI: 0.401–0.539). The distribution was not explained by bracket-base design alone. ARI score 1 predominated in the MP group (81.8%), whereas ARI scores 2 and 3 predominated in the integral-base groups. This pattern suggests that both bracket-base morphology and primer-coverage condition may influence the locus of bond failure. Recent studies likewise show that changes in bracket-base geometry can be accompanied by changes in ARI as well as SBS [16,17]. The ARI findings should therefore be interpreted together with SBS rather than as an isolated outcome. Importantly, the present study did not directly characterize the microscopic failure interface or primer distribution; consequently, mechanistic explanations regarding where and why failure shifted should be regarded as hypotheses rather than established mechanisms.

From a clinical perspective, statistical superiority in SBS does not necessarily indicate a preferable bonding condition. Mean SBS values in all six groups (12.66–20.89 MPa) exceeded the historically cited minimum range of approximately 6–8 MPa for orthodontic bonding [3]; a recent bracket-base study likewise emphasized that designs with SBS values above this range may all provide clinically adequate retention [16]. More broadly, a 2025 systematic review reported lower clinical bracket-failure rates when a conventional orthodontic primer step was used than when primer was omitted [18], supporting the practical importance of reliable bonding, although that evidence does not specifically evaluate metal primer applied to bracket bases. Excessively high bond strength may be undesirable if it complicates debonding or shifts failure toward the enamel. Therefore, the clinically relevant objective is to balance adequate retention, a manageable failure pattern, and enamel preservation rather than simply maximize SBS.

Despite the differences in ARI distribution, EDI scores did not differ significantly among groups. Under the conditions of this study, the observed differences in SBS and failure pattern therefore did not translate into a detectable difference in enamel material loss at the stereomicroscopic level. This finding does not establish equivalence with respect to subtle enamel damage, because the binary EDI used here records only visible material loss and may miss microcracks, changes in surface roughness, or other microscopic defects. Recent thermocycling research has evaluated enamel microcracks using more sensitive microscopic approaches and highlighted the need to consider enamel response together with bond-strength behavior [22]. More sensitive methods, such as scanning electron microscopy or quantitative surface profilometry, are therefore warranted in future studies.

This study has several limitations. It was conducted in vitro and used a unidirectional shear test, which cannot fully reproduce the thermal, mechanical, and moisture-related challenges of the oral environment. Only one metal primer and one adhesive system were tested; therefore, the findings may be specific to this material combination and should not be generalized to other primer–adhesive systems without further testing. The exclusive use of maxillary premolars improved substrate consistency but limits extrapolation to other tooth types. Bracket placement was performed using a consistent operator technique, but no dedicated positioning jig was used, and adhesive-layer thickness was not measured quantitatively. The binary EDI is a relatively coarse measure of enamel damage. The partial primer-coverage area was defined by visual estimation and standardized using a 1 mm microbrush applied perpendicular to the central region of the bracket base rather than by direct geometric measurement; therefore, minor variation in the exact treated surface area cannot be excluded. ARI/EDI scoring and ImageJ measurements were performed by a single examiner, but formal intra-examiner repeatability and inter-examiner reliability testing were not performed; this should be considered when interpreting the scoring outcomes. In addition, the six-group ARI contingency table contained several cells with expected frequencies below five. Although a Monte Carlo conditional test confirmed the significance of the overall association, the sparse distribution of some ARI categories remains a limitation for detailed category-level inference. Future studies should include long-term aging, cyclic loading, moisture contamination, additional primer and adhesive systems, multiple tooth types, quantitative control of adhesive thickness, formal scoring-reliability assessment, direct microscopic or chemical characterization of the primer–adhesive interface, larger samples for categorical failure analysis, and more sensitive enamel-assessment methods [4,22].

## 5. Conclusions

Within the limitations of this in vitro study, bracket-base design and primer coverage significantly influenced SBS, and the significant interaction indicated that the effect of metal primer depended on bracket-base design. Mesh-base brackets showed higher SBS overall than integral-base brackets. Within the mesh-base system, partial primer coverage produced significantly higher SBS than the control, whereas no significant within-design pairwise differences were detected among the integral-base groups, despite the numerically highest SBS with full coverage. ARI distributions also differed significantly across the six experimental groups, indicating that adhesive-remnant patterns varied according to the combined experimental conditions rather than bracket-base design alone. No significant difference in stereomicroscopically detectable enamel material loss was observed. Because all group means were already above the commonly cited minimum SBS range, these findings should not be interpreted as favoring the highest bond strength per se; clinically, an adequate and predictable bond must be balanced against safe debonding and enamel preservation. Further studies are needed to confirm these findings under clinically representative conditions and with additional primer, adhesive, bracket, and tooth systems.

## Figures and Tables

**Table 1:** Definition of the six experimental groups by bracket base design and metal-primer coverage.

Code	Base Design	Metal-Primer Coverage	n
MC	Mesh	None (control)	22
MF	Mesh	Full-base coverage	22
MP	Mesh	Partial (central) coverage	22
IC	Integral	None (control)	22
IF	Integral	Full-base coverage	22
IP	Integral	Partial (central) coverage	22

Note: Group codes: M = mesh, I = integral; C = control, F = full coverage, P = partial coverage.

**Table 2:** Descriptive statistics for shear bond strength (SBS, MPa) by group.

Base	Group	n	Mean	SD	SE	95% CI	Min.	Max.
Mesh	MC	22	15.68	7.86	1.68	12.19–19.16	4.07	36.74
	MF	22	18.38	7.01	1.49	15.27–21.48	5.61	36.52
	MP	22	20.89	5.00	1.07	18.67–23.11	12.87	32.45
	Mesh total	66	18.31	6.96	0.86	16.60–20.03	4.07	36.74
Integral	IC	22	13.04	5.07	1.08	10.80–15.29	4.51	22.86
	IF	22	17.11	4.29	0.91	15.20–19.01	9.73	24.05
	IP	22	12.66	4.65	0.99	10.60–14.72	6.72	21.76
	Integral total	66	14.27	5.03	0.62	13.03–15.51	4.10	24.05

SBS, shear bond strength (MPa); SD, standard deviation; SE, standard error; CI, confidence interval for the mean. Group codes: first letter denotes bracket base (M = mesh, I = integral); C = control (no metal primer); F = full (complete) primer coverage of the base; P = partial (central) primer coverage.

**Table 3:** Pairwise comparison of shear bond strength between experimental groups.

Comparison	Mean Difference (I − J), MPa	95% CI	Tukey-Adjusted *p*-Value
MC vs. MF	−2.700	−7.757 to 2.357	0.636
MC vs. MP	−5.215	−10.272 to −0.158	0.039
MC vs. IC	2.632	−2.426 to 7.689	0.661
MC vs. IF	−1.432	−6.489 to 3.626	0.963
MC vs. IP	3.013	−2.044 to 8.071	0.518
MF vs. MP	−2.515	−7.572 to 2.542	0.703
MF vs. IC	5.332	0.274 to 10.389	0.032
MF vs. IF	1.268	−3.789 to 6.326	0.978
MF vs. IP	5.713	0.656 to 10.771	0.017
MP vs. IC	7.847	2.789 to 12.904	<0.001
MP vs. IF	3.783	−1.274 to 8.841	0.262
MP vs. IP	8.228	3.171 to 13.286	<0.001
IC vs. IF	−4.064	−9.121 to 0.994	0.192
IC vs. IP	0.381	−4.676 to 5.439	1.000
IF vs. IP	4.445	−0.612 to 9.502	0.120

Note: Mean differences are expressed as group I minus group J. Positive values indicate higher SBS in the first-listed group. *p*-values and 95% confidence intervals are based on Tukey HSD adjustment for multiple comparisons. Statistical significance was set at *p* < 0.05. Group codes: MC = mesh control; MF = mesh full primer coverage; MP = mesh partial primer coverage; IC = integral control; IF = integral full primer coverage; IP = integral partial primer coverage.

**Table 4:** Distribution of adhesive remnant index (ARI) scores across the six experimental groups.

Base	Group	ARI 0, n (%)	ARI 1, n (%)	ARI 2, n (%)	ARI 3, n (%)	Total
Mesh	MC	6 (27.3)	5 (22.7)	9 (40.9)	2 (9.1)	22 (100.0)
	MF	7 (31.8)	8 (36.4)	7 (31.8)	0 (0.0)	22 (100.0)
	MP	4 (18.2)	18 (81.8)	0 (0.0)	0 (0.0)	22 (100.0)
Integral	IC	0 (0.0)	3 (13.6)	14 (63.6)	5 (22.7)	22 (100.0)
	IF	0 (0.0)	6 (27.3)	14 (63.6)	2 (9.1)	22 (100.0)
	IP	0 (0.0)	2 (9.1)	12 (54.5)	8 (36.4)	22 (100.0)

ARI, adhesive remnant index (0 = no adhesive on the tooth; 1 ≤ 50% adhesive on the tooth; 2 ≥ 50% adhesive on the tooth; 3 = all adhesive on the tooth). Values are counts with percentages within each experimental group. Overall six-group association: Pearson χ^2^(15) = 75.24, *p* < 0.001; Monte Carlo conditional *p* < 0.001 (100,000 random tables with fixed marginal totals); Cramér’s V = 0.436 (95% bootstrap CI: 0.401–0.539). Group codes: MC = mesh control; MF = mesh full primer coverage; MP = mesh partial primer coverage; IC = integral control; IF = integral full primer coverage; IP = integral partial primer coverage.

**Table 5:** Distribution of EDI scores across the six experimental groups (chi-square test).

Base	Group	EDI 0, n (%)	EDI 1, n (%)	*p*
Mesh	MC	14 (63.6)	8 (36.4)	0.756
	MF	11 (50.0)	11 (50.0)	
	MP	13 (59.1)	9 (40.9)	
Integral	IC	14 (63.6)	8 (36.4)	
	IF	11 (50.0)	11 (50.0)	
	IP	15 (68.2)	7 (31.8)	

EDI, enamel damage index (0 = no detectable material loss on the buccal enamel surface; 1 = detectable material loss after debonding and adhesive clean-up). The *p* value is from the chi-square test across the six groups.

## Data Availability

The raw data supporting the conclusions of this study are available from the corresponding author upon reasonable request. A minimal dataset has been provided to the Editorial Office for editorial evaluation. Raw data are provided as Supplementary Materials (File S1).

## Supplementary Materials

The following supporting information can be downloaded at: https://www.mdpi.com/article/10.3390/dj14090581/s1, File S1: Raw data underlying the findings reported in this study.
